# Form meets function meets fashion: convergent drawing of multimaterial fibers

**DOI:** 10.1093/nsr/nwae111

**Published:** 2024-03-22

**Authors:** John Ballato

**Affiliations:** Department of Materials Science and Engineering, Clemson University, USA

Fibers are ubiquitous material forms that permeate a great many aspects of daily lives from silica glass optical fibers that enable this Information Age to polymer fibers used in our apparel to soft glass fibers used for thermal insulation and mechanical strengthening.

As societal needs progress, technological solutions advance, often in the form of unifying existing but individualized approaches into integrated versions. Historically, the transition from vacuum tubes to solid state transistors, and then to integrated circuits (and then to many millions of them on a single minute chip) is a useful example. In fibers, those noted singular solutions, e.g. optical fibers for communication and polymer fibers for apparel, began to coalesce about two decades ago with the nascent development of ‘multimaterial’ fibers. These fibers integrated polymers, glasses, and metals into a single thermally drawn structure to form fabrics that ‘see, hear, sense and communicate [1].’

In those intervening decades, such multimaterial, multifunctional fibers and fabrics have grown impressively in both the range of materials and complexities of their interconnections and applications [2,3]. However, often overlooked in the sexiness of science is the earnestness of engineering.

There are many ways to make a fiber. There are, however, only a few ways to make a practical fiber, and lots of it. Though spinning works well where dimensional control is not so critical, thermal drawing reigns supreme when control is needed and, as is the case here, highly dissimilar materials need to be co-processed. To-date, thermal co-drawing of very different types of materials, e.g. polymers, metals, and glasses, has generally necessitated doing so at relatively low temperatures. This precludes then a wide variety of high-performance materials, including important semiconductors such as Si and Ge, upon which virtually all microelectronics and optoelectronics are based.

This is where the recent work by Wang *et al.* [4] makes important contributions to the utility of multimaterial fibers and smart fabrics made therefrom. Though the thermal drawing of glass-clad Si and Ge semiconductor fibers was first performed in the late 2000s [5,6], the primary focus of those fibers has since been towards optical applications [7]. Also realized previously was that cladding glasses tailored for the thermomechanical properties of the semiconductor core would be critical to preserving high-quality semiconductors over long (>100 m) continuous lengths [8]. Following a detailed analysis of capillary instabilities and thermomechanical challenges, Wang *et al.* [4] employ the scalable molten core method to fabricate long lengths of glass-clad semiconductor fibers, removing the glass, which then affords high-quality semiconductor wires that can then be ‘convergently drawn’ with conductive polymers (e.g. carbon-filled polycarbonate, CPC) and metals (e.g. copper or tungsten), clad in a transparent polycarbonate cladding.

**Figure 1.**
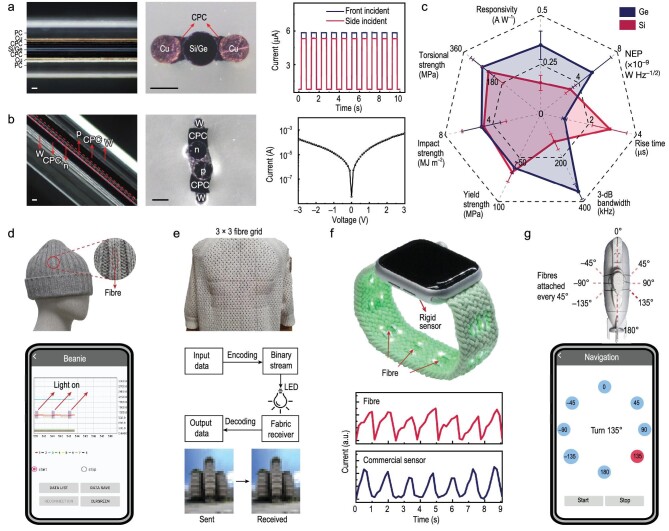
(a) Left and middle: single-core optoelectronic fiber. Left and middle: optical images of the side-view (left) and cross-section (middle) of the single-core optoelectronic fiber, where the core semiconductor is connected to each copper electrode through a layer of CPC. Right: the resulting optoelectronic fiber shows a pseudo-omnidirectional response that maintains sensitivity for different directions. PC, polycarbonate. Scale bars, 50 μm. (b) Left and middle: optical images of the side-view (left) and cross-section (middle) of the dual-core p–n junction fiber. Right: the *I*–*V* characteristic of the p–n junction fiber. Scale bars, 50 μm. (c) Overall performance evaluation of the resulting optoelectronic fibers. The noise equivalent power (NEP) value of the Si optoelectronic fibers is amplified by a factor of ten for visualization. For measurement of responsivity and NEP, *n* = 9 for all cases. For other measurements, *n* = 6 for all cases. All data are presented as mean ± s.d. (d) Top: the functional beanie used in the demonstration of outdoor use as an assistive wearable device. The interface board was placed inside the beanie tip. Bottom: the signal received by the beanie is visualized in a mobile application. (e) Top: a functional sweater for indoor Li-Fi communication system. Middle: block diagram of receiving data via the functional sweater. Bottom: a photo of a building (the Learning Hub at Nanyang Technological University) was sent and received via the sweater. (f) Top: a watchband measures heart pulses via photoplethysmography. Traditionally, a rigid sensor is installed on the backside of the watch. The optoelectronic fibers are woven into the watchband, turning the watchband into a flexible and conformal sensor. Bottom: a comparison of the measured pulse between the fiber and the commercial sensor. (g) Top: fiber receiver array for underwater visible-light communication system. Fibers were conformally attached to the mini-submarine every 45°, dividing the circumference of the mini-submarine into 8 sections, where each fiber represented a specific angle. Bottom: the command line in the mobile application shows ‘Turn 135°’, when the fiber at 135° receives the signal. Figure and caption reproduced as printed in Ref. [4] under Creative Commons license 4.0.

From this combined process, multimaterial and multifunction fibers were realized based on higher temperature and more commercially accepted phases than have been previously employed. With them, Wang *et al.* fabricated and tested an impressive array of important electronic and optoelectronic functions including back-to-back Schottky contacts that enabled a pseudo-omnidirectional response to incident light and dual-core p–n junction fibers. These useful in-fiber devices were then fashioned into assistive wearable structures including a functional beanie demonstrating outdoor use, a functional sweater for indoor Li-Fi communication, and a watchband that measured heart pulses via photoplethysmography (PPG), see Fig. 1.

As the thermal drawing of glass-clad semiconductor fibers enjoys its 15-year anniversary [5,6], creative and innovative work like that by Wang *et al.* significantly broadens and advances the field. Indeed, this work shows that we are only scratching the surface of what is possible with multifunctional, multimaterial fibers and wearable structures and sensors based on them.
